# Gut Microbiomes of the Eastern Oyster (*Crassostrea virginica*) and the Blue Mussel (*Mytilus edulis*): Temporal Variation and the Influence of Marine Aggregate-Associated Microbial Communities

**DOI:** 10.1128/mSphere.00730-19

**Published:** 2019-12-11

**Authors:** Melissa L. Pierce, J. Evan Ward

**Affiliations:** a Department of Marine Sciences, University of Connecticut at Avery Point, Groton, Connecticut, USA; University of Wisconsin—Madison

**Keywords:** oyster, mussel, microbiome, aggregate, gut, bivalve, bacteria, 16S rRNA gene

## Abstract

Gut microbial community structure was evaluated for two species of bivalve molluscs, the eastern oyster (Crassostrea virginica) and the blue mussel (Mytilus edulis) collected from Long Island Sound, Connecticut, over the course of a year. These bivalves utilize a shared feeding mechanism, which may result in similar gut microbial communities. Their particle diet, marine aggregates, and surrounding environment, aggregate-free seawater (AFSW), were also collected for comparison. Due to the suspension-feeding activities of bivalves, the potential for aggregate- and AFSW-associated microbiota to influence their microbial communities may be significant. Both taxonomic and functional diversity of the samples were assessed. 16S rRNA gene amplicon sequencing indicated that oysters and mussels maintained similar, but not identical, gut microbiomes, with some temporal variation. Throughout the year, bivalve species had gut microbial community compositions that were more similar to one another than to aggregates. Within a month, bivalves shared on average a quarter of their total operational taxonomic units (OTUs) with each other and a 10th of their total OTUs with aggregates. During months with warm water temperatures, individuals within each of the four sample types had similar alpha diversity, but again, temporal variation was observed. On a functional level, bivalve gut microbial communities exhibited variation attributed to host species and season. Unlike oysters, mussel gut bacterial communities maintained high richness and evenness values throughout the year, even when values for the particle diet and AFSW were reduced. Overall, a core gut bivalve microbiome was present, and it was partially influenced by the marine aggregate microbial community.

**IMPORTANCE** This work investigates the influence that extrinsic factors, diet, and the environment can have on the microbiomes of shellfish. Over the course of a year, the gut microbial communities of two species of bivalves, oysters and mussels, held under identical conditions in coastal marine waters were compared. While the mussels and oysters harbored gut microbial communities with similar composition, on a functional level, they exhibited species and temporal variation. These results indicate that intrinsic factors influence the bivalve microbiome, resulting in species variability, even when environmental conditions, feeding mechanism, and particle diet are constant. Seasonal and multispecies comparisons for bivalve-associated microbial communities are rare, and we believe this research represents an important contribution. The results presented here advance our understanding of the symbiotic interactions between marine invertebrates, the microbial communities they harbor, and the environment.

## Abstract (precis)

This work investigates the influence that extrinsic factors, diet, and the environment can have on the microbiomes of shellfish. Over the course of a year, the gut microbial communities of two species of bivalves, oysters and mussels, held under identical conditions in coastal marine waters were compared. While the mussels and oysters harbored gut microbial communities with similar composition, on a functional level, they exhibited species and temporal variation. These results indicate that intrinsic factors influence the bivalve microbiome, resulting in species variability, even when environmental conditions, feeding mechanism, and particle diet are constant. Seasonal and multispecies comparisons for bivalve-associated microbial communities are rare, and we believe this research represents an important contribution. The results presented here advance our understanding of the symbiotic interactions between marine invertebrates, the microbial communities they harbor, and the environment.

## INTRODUCTION

Research conducted on animal microbiomes demonstrates a strong interaction between hosts and the microbial communities they harbor (1–5). These interactions span a variety of processes, from digestion and nutrient absorption to immune response and development, and may play a major role in host homeostasis (5–8). How the gut microbial community is modulated, however, is still poorly understood, but it could include both extrinsic (e.g., environment, diet) and intrinsic (e.g., host immune system, physiology, life stage) components (4, 9–11).

Although mammals have been most frequently studied (2), research focused on understanding invertebrate gut-microbiome interactions has grown. In the fruit fly Drosophila melanogaster, the gut microbiota have been found to be affected by environment and diet, with differences seen between laboratory-reared and wild-type flies (12). Similarly, annelid worms showed a rapid and homologous change in the microbiota in response to feeding after starvation; however, they also harbored bacteria not found in their diet or bedding (13). In the aquatic environment, different species of *Hydra* had different bacterial communities even when cultured under identical conditions (14). Laboratory-reared and wild-type *Hydra* of the same species shared similar microbiota, supporting the idea that the host imparts some control on the microbiome (14, 15). Marine sponges, although they lack true tissues or a gut, contain bacterial assemblages that are distinct from the surrounding seawater and highly conserved among species over time and space (16–18). Corals also appear to maintain species-specific gut microbiomes, even when separated geographically (19–21). Abalone, a gastropod mollusc, demonstrated changes in biodiversity of their gut microbiota when different diets were applied (22). Multiple studies on bivalve molluscs showed that gut microbiota of several oyster species were influenced by life stage, host taxonomy, and environmental parameters (i.e., temperature) (23–25). Specifically, the gut communities of the eastern oyster (Crassostrea virginica) were not significantly different from one another when separated geographically, but they were characterized by seasonal, temperature-dependent differences (25).

In all cases, both intrinsic and extrinsic factors are likely at play, with the environment as well as the host asserting control on the microbiome composition. In the aquatic environment, one of the least studied parameters is the influence of diet. For sessile suspension-feeding bivalves such as oysters and mussels, direct interactions with the suspended particle diet could affect their gut microbiomes. In particular, the presence of particle aggregations could impact types and numbers of bacteria that are captured and ingested by bivalves. Marine aggregates (also known as marine snow and flocs) are a natural collection of living and nonliving materials that have agglomerated through physical, chemical, and biological processes (26–30). Aggregates are ubiquitous in the marine environment and have a complex three-dimensional structure that is physically and chemically distinct from the surrounding seawater (31–33). These microhabitats provided an enriched nutrient environment for microbes (34, 35), and they are an area of high biomass, productivity, and diversity compared to seawater (36–41), with enrichment factors often on the order of 100 to 1,000 (37, 42). Community structure, diversity, enzyme activity, and metabolism of the marine aggregate-associated microbes often differ significantly from that of the water and free-living (unattached) microbial communities (30, 36, 38, 43–47).

The capture and ingestion of particles by bivalves are largely dependent on size, with capture efficiency increasing nonlinearly with increasing particle size (48–50). Particles of <1.5 μm, such as individual bacteria, are captured by oysters and mussels at <25% efficiency (50–54), whereas microbes associated with aggregates of >10 μm are captured at >90% efficiency. At an average size of 500 μm, marine aggregates represent a vehicle for enhanced encounter and uptake of microbes by bivalves (39, 55, 56).

The goal of the current study was to better understand the dynamic relationship between bacterial communities of marine aggregates, aggregate-free seawater (AFSW), and the bivalve gut. A seasonal study of the suspended-particle diet and two species of bivalves was conducted to assess both extrinsic (environmental) and intrinsic (bivalve species) factors that could mediate the microbial community structure and functional diversity of each microenvironment (aggregates, aggregate-free seawater, bivalve gut). Specifically, this project was designed to address the hypothesis that mussels and oysters would harbor similar gut microbial communities because these bivalves utilize a shared feeding mechanism. Additionally, the components of their particle diet (marine aggregates) and environment (seawater) may impart an influence on the composition of the bivalve gut microbiome.

## RESULTS

### 16S rRNA gene amplicon sequencing.

A total of 2.3 million sequences were analyzed from 36 samples, with a median of 59,045 reads per sample. After filtering, across all sample types and dates, a total of 1,113 operational taxonomic units (OTUs) were observed. Mussel gut samples had 989 total OTUs, oyster gut samples had 781 OTUs, marine aggregate samples had 1,004 OTUs, and aggregate-free seawater (AFSW) had 861 OTUs.

**FIG 1:**
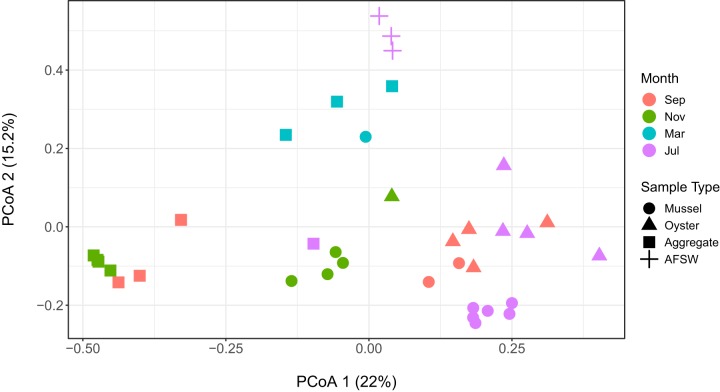
Dissimilarity between samples was measured using the Bray-Curtis distance metric. Each sample type is represented by a unique symbol, and each month (September, November, March, and July) is represented by a unique color. The percent variation explained by each axis is shown in parentheses. Samples cluster according to sample type and month, with some overlap between the two bivalve species. A reduced number of replicates for some samples, in some months, resulted from a failure of samples to amplify.

**(i) Microbial community composition and diversity.** Principal coordinate analysis (PCoA) using the Bray-Curtis metric revealed that sequences grouped by sample type and month (Fig. 1). During the month of March, however, marine aggregate samples grouped separately from those of September, November, and July. Within each sample type, individual samples clustered by month. Permutational multivariate analysis of variance (PERMANOVA) results supported these trends, with the factors month (*P* < 0.001, *R*^2^ = 0.25, Pseudo-*F *= 3.4) and sample type (*P* < 0.001, *R*^2^ = 0.31, Pseudo-*F *= 5.4) contributing significantly to the variation observed among the microbial communities. Overlap between the two bivalve species was evident, especially in the month of September, with mussel and oysters clustering together, and variation in the gut microbiome was not attributable to the species of bivalve (PERMANOVA; *P* > 0.05).

**FIG 2:**
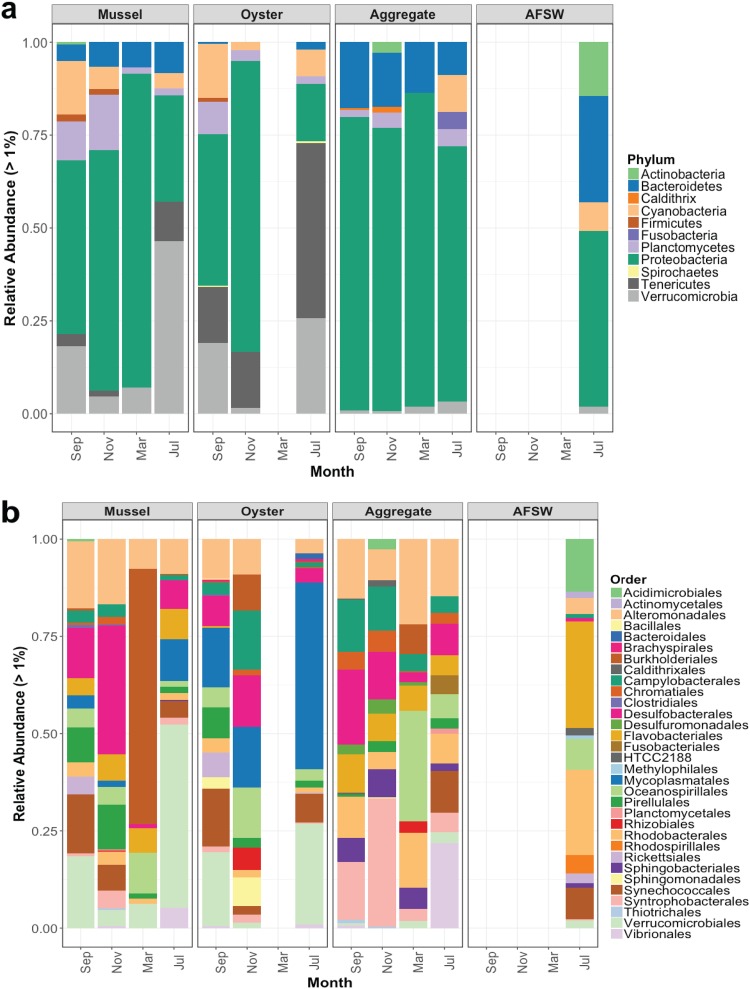
Community composition of each sample type (mussel gut, oyster gut, aggregate, aggregate-free seawater [AFSW]) by phylum (a) and order (b). The relative abundance of each taxonomic group >1% of the total is listed.

**FIG 3:**
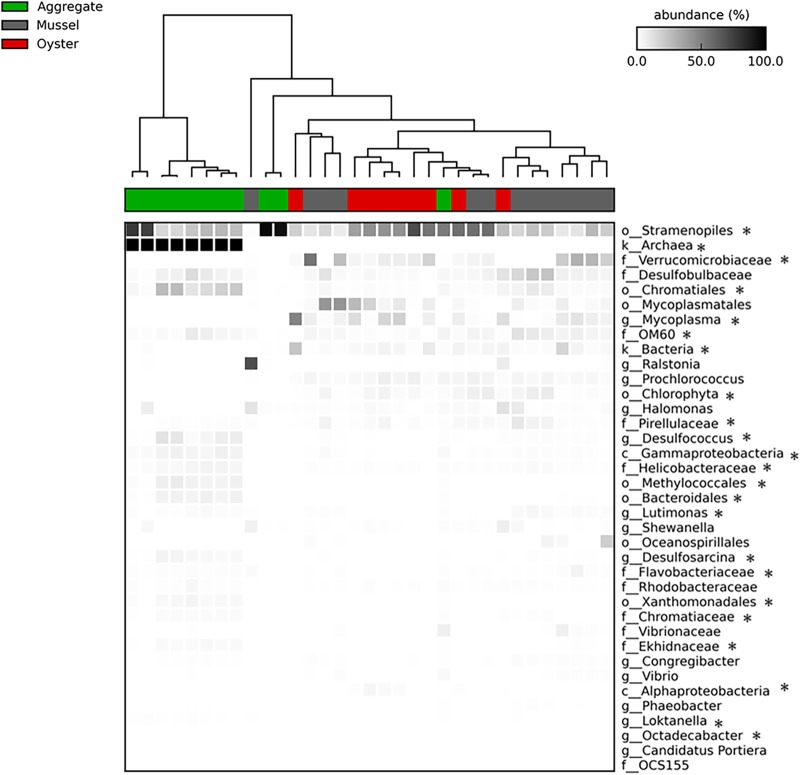
Heat map showing the abundances of different taxa, using the lowest taxonomic level available. Lowercase letters designate taxonomic level (o for order, f for family, g for genus, k for kingdom, etc.). Significant differences in abundance between sample types are indicated by an asterisk next to that taxonomic group (one-way ANOVA, *P* < 0.05). The number of samples follow: mussels (*n* = 13), oysters (*n* = 9), and aggregates (*n* = 11).

The most abundant phyla across sample types were the *Proteobacteria*, *Tenericutes*, *Verrucomicrobia*, *Bacteroidetes*, *Cyanobacteria*, *Plantomycetes*, *Actinobacteria*, *Firmicutes*, *Cladithrix*, and *Fusobacteria*. (Fig. 2a). Overall, bivalves contained significantly higher abundances of *Verrucomicrobia* and *Tenericutes* but significantly fewer *Bacteroidetes* than marine aggregates (Fig. 3). Temporally, marine-aggregate communities changed little at the phylum level. Bivalve samples demonstrated seasonal variation in the proportion of abundant phyla, but not a change in which phyla were the most abundant. Greater temporal variability could be seen at lower bacterial taxonomic levels within each of the sample types (Fig. 2b).

As determined by SIMPER (similarity percentage) analysis, microbial communities within each sample type were highly similar (>78%) both within a month and when all months were combined (see Table S1 in the supplemental material). Dissimilarity between sample types was low, and oysters and mussels had the lowest dissimilarity to each other (18%) than either had to marine aggregates (27 to 32%). The highest dissimilarities between months were found with comparisons to March. For all sample types, the most variation was always explained by *Cyanobacteria* and *Proteobacteria*. *Cyanobacteria* were the largest driver of dissimilarity between sample types, except for oyster versus AFSW dissimilarities which were driven by *Tenericutes*, and mussel versus AFSW dissimilarities which were driven by *Verrucomicrobia*. No bivalve samples contained *Archaea*. Dissimilarity levels of individual oysters between months ranged from 13 to 18%, individual mussels ranged from 15 to 25%, and individual aggregates ranged from 13 to 33%.

10.1128/mSphere.00730-19.3 TABLE S1 SIMPER analysis for 16S rRNA sequencing data (phylum level) and for EcoPlate absorbance data. Download Table S1, PDF file, 0.2 MB. Copyright © 2019 Pierce and Ward. 2019 Pierce and Ward This content is distributed under the terms of the Creative Commons Attribution 4.0 International license.

**FIG 4:**
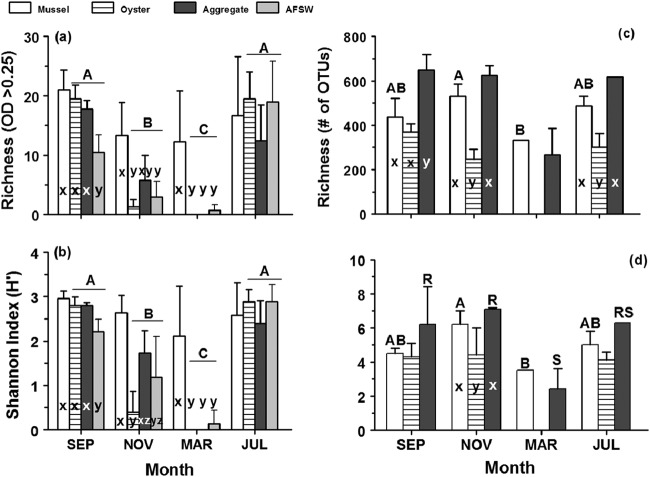
(a and b) Richness of carbon sources utilized by heterotrophic microbial communities (optical density of >0.25 at 168-h reading) (a) and Shannon diversity index (b) by sample type (mussel gut, oyster gut, aggregate, aggregate-free seawater [AFSW]) and month (EcoPlates). Effects of month and sample type were analyzed by means of two-way ANOVA. (c and d) Richness (number of OTUs observed) (c) and Shannon diversity index (d) by sample type and month (16S rDNA). Individual one-way ANOVAs were run due to interaction effects. 16S rDNA data from AFSW samples were not used in the analyses because only July samples amplified. In all panels, different capital letters represent significant differences between months, whereas significant differences between sample types within a month are indicated by lowercase letters (Tukey’s HSD, *P* < 0.05). Where there are no letters, no significant effects were observed. Data are presented as means plus standard deviations (SD) (error bars) with *n* = 4 to 6 (EcoPlates) and *n* = 1 to 6 (16S rDNA) per sample type per month.

Shannon diversity index values were similar across the months September, November, and July for all sample types (Fig. 4d; one-way analysis of variance [ANOVA], *P* > 0.05). In March, significant differences were seen for marine aggregate and mussel samples (one-way ANOVA, *P* < 0.05). Similar results were seen for the number of OTUs observed for each sample type by month. No significant differences were observed for marine aggregates, but mussel samples in March decreased significantly compared to November (Fig. 4c). Marine aggregates had significantly higher numbers of OTUs compared to oysters in September, November, and July.

**FIG 5:**
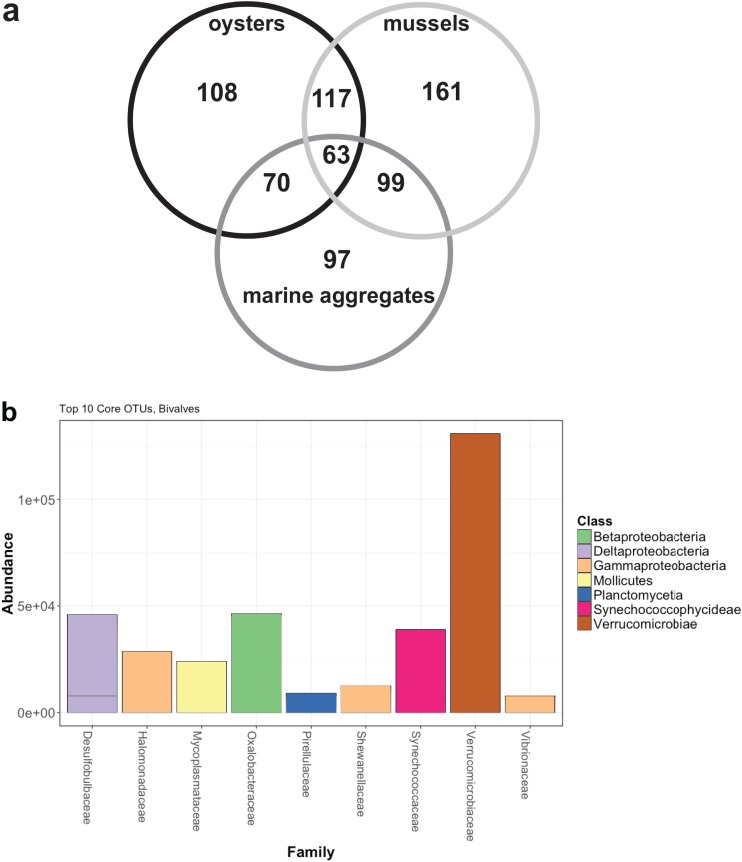
(a) Venn diagram of core OTUs for three of the sample types (mussel gut, oyster gut, aggregate) from all months. Core OTUs are defined here as those shared by 95% of samples. AFSW samples were not used in the analyses because only July samples amplified. Sample sizes used are as follows: mussels (*n* = 13), oysters (*n* = 9), and aggregates (*n* = 10). (b) Abundance of the top 10 core OTUs shared by mussels and oysters from all months.

**(ii) Core microbiome and shared OTUs.** Core OTUs (defined as those OTUs shared across 95% of samples analyzed) were evident among and within sample types (Fig. 5). Mussels had the highest number of OTUs in their year-round core microbiome at 161 OTUs (16.2% of total OTUs sequenced), followed by oysters at 108 OTUs (13.8% of total) and marine aggregates at 97 OTUs (9.6% of total). Oyster and mussel samples from all seasons shared the highest percentage of their core microbiome (117 OTUs [11 to 15%]). In contrast, each bivalve shared 8 to 9% of their core microbiome with marine aggregates. Only AFSW samples from July were greater, and overall comparison with other sample types was not possible.

Within a month, mussel gut samples from September shared 60.6% of total OTUs sequenced, November animals shared 50.7%, and July animals shared 39%. Comparison within the month of March was not possible due to *n* = 1 (see Fig. S1a in the supplemental material). Oyster gut samples from September shared 40.5% of the total OTUs sequenced, and 31.8% from all July animals (Fig. S1b). Within each month, both bivalves shared on average 23.4% of the total OTUs. Some of the most abundant shared OTUs in the bivalve gut core were the Deltaproteobacteria (19 OTUs), sulfur metabolizers belonging to the *Desulfobulbaceae*, *Desulfobacteraceae*, and *Desulfuromonadaceae* families. Also abundant were the *Verrucomicrobia* (10 OTUs) and 37 OTUs belonging to the *Gammaproteobacteria*, mostly those in the orders *Chromatiales*, *Oceanospirillales*, and *Alteromonadales* (*OM60* and *Shewanellaceae* families). Particularly notable was the presence of three *Phaeobacter* OTUs in the bivalve core, a genus containing a known oyster probiotic shown to protect the host against *Vibrio* and *Roseovarius* pathogens (57).

10.1128/mSphere.00730-19.1 FIG S1 Venn diagrams showing number of OTUs within the mussel gut (a), oyster gut (b), and aggregate (c). Values in smaller circles represent the core OTUs for a given month, whereas values in larger circles represent the total number of OTUs sequenced. Core OTUs are defined here as those shared by 95% of samples. Values between months are those OTUs shared by 95% of samples out of the total number of OTUs for all overlapping samples. The months (September, November, March, and July) are indicated. Download FIG S1, PDF file, 0.4 MB. Copyright © 2019 Pierce and Ward. 2019 Pierce and Ward This content is distributed under the terms of the Creative Commons Attribution 4.0 International license.

For marine aggregates, September samples shared 72.5% of the total OTUs sequenced, November samples shared 59.8% of OTUs, and March samples shared 27.1% of OTUs (Fig. S1c). The most abundant OTUs in the core across all months belonged to the *Bacteroidetes* (13 OTUs) and the *Alphaproteobacteria* (10 OTUs), Deltaproteobacteria (17 OTUs), and *Gammaproteobacteria* (42 OTUs). In contrast to the bivalves, there were no OTUs belonging to the *Verrucomicrobia* in the core aggregate microbiome, although these OTUs were present in some aggregate samples.

The numbers of shared OTUs among mussel, oyster, and aggregate samples from individual months were compared to better understand the role of the particle diet. In September, bivalves shared 96 OTUs with marine aggregates, accounting for 9.7% of the total 994 OTUs sequenced from that month. November and July were consistent with this trend, with 10.5% and 14.3% of the total OTUs observed shared among all three sample types, respectively. Those OTUs that were consistently shared among bivalves and aggregates throughout the year belonged to the *Gammaproteobacteria* (*Halomonadaceae*, *Shewanella*, *OM60*) and Deltaproteobacteria (*Desulfobulbaceae*, *Desulfobacteraceae*) as well as the *Cyanobacteria* (*Synechococcaceae*) and *Planctomycetes* (*Pirellulaceae*) phyla. Temporal variation was seen, with *Rhodobacteraceae* OTUs being shared among all three sample types in September, *Verrucomicrobiaceae* in September and July, and *Vibrionaceae* and *Flavobacteriaceae* in November and July.

### Community-level physiological profiling—EcoPlates. (i) SIMPER analysis.

Each sample type had moderate to low similarity among individuals when all months were considered. Seasonal trends were seen when individual months were analyzed (Table S1), with the highest similarities occurring in September and July and the lowest similarities occurring in November and March. Overall, AFSW samples were the least similar to one another, and marine aggregate samples had a lower dissimilarity to AFSW than either bivalve had to AFSW. Mussel, oyster, and marine aggregate samples had comparable levels of dissimilarity to each other (62 to 64%).

Comparison of mussel samples across months revealed that carbohydrates, amino acids, and polymers explained 36.4%, 25.7%, and 22.3% of the variation, respectively, whereas the carbohydrate *N*-acetyl-d-glucosamine and the polymer Tween 40 contributed the most. Dissimilarity between months varied between 30 and 50%. For oysters, polymers explained 41.6% of the variation, and carbohydrates explained another 29.5%, with the polymers Tween 40 and α-cyclodextrin contributing the most. September versus July had the lowest dissimilarity level (23%), while all other comparisons ranged in dissimilarity from 72 to 80%. For marine aggregates, polymers explained 42.8% of the variation, whereas carbohydrates explained another 29.5%, with the polymer glycogen and the carbohydrate d-cellobiose responsible for the most variability. September and July had the lowest dissimilarity level (26%), and all other comparisons varied in dissimilarity (53 to 73%). For AFSW samples, carbohydrates, polymers, and amino acids explained 38%, 30%, and 20% of the variation, respectively, whereas the carbohydrates d-cellobiose and *N*-acetyl-d-glucosamine contributed the most. September and July had the lowest dissimilarity level (40%), and all other comparisons ranged in dissimilarity from 72 to 84%.

**(ii) Richness, Shannon diversity index, and evenness.** All metrics were significantly affected by month and sample type (two-way ANOVA, *P* < 0.05, df = 79). Due to significant interaction effects (two-way ANOVA, *P* < 0.05), the model was broken into individual one-way ANOVA and rerun. The richness (number of carbon sources utilized [*S*]) and Shannon diversity index of heterotrophic microbial communities changed seasonally for oyster, marine aggregate, and AFSW samples (Fig 4a and b). This trend was not seen with the gut microbial communities of mussels, whose richness and Shannon index did not vary temporally. For both metrics, post hoc analysis (Tukey’s honestly significant difference [HSD] test) showed significant differences among sample types within each month except for July (*P* < 0.05; Fig. 4a and b). Mean evenness values are listed in Table 1, and corresponding temperatures for sampling time points are listed in Table 2.

**TABLE 1:** EcoPlate evenness values for sample types by month.

Sample	*n* per mo	EcoPlate evenness value (mean ± SD)			
		September	November	March	July
Mussel	6	0.97 ± 0.007	0.96 ± 0.021	0.81 ± 0.40	0.99 ± 0.004
Oyster	6	0.94 ± 0.057	0.45 ± 0.50	0	0.98 ± 0.003
Aggregate	4	0.97 ± 0.007	0.96 ± 0.025	0	0.98 ± 0.008
AFSW	4	0.95 ± 0.026	0.66 ± 0.45	0.21 ± 0.42	0.97 ± 0.004

**TABLE 2:** Characteristics of environmental samples for each month^*a*^

Month	TSS (mg liter^−1^)	POM (%)	PIM (%)	No. of aggregates ml^−1^	Temp (°C)	Salinity
September	9.5 ± 4.8	21.8 ± 3.3	78.2 ± 3.3	15.9–62.5	17.8–18.4	29.2–29.5
November	7.1 ± 2	17.5 ± 2.5	82.5 ± 2.5	N/A	8.9–9.3	30–30.2
March	8.1 ± 1.5	19.2 ± 9	80.8 ± 9	8.9–9.1	2.2–3.1	28.1– 29.2
July	11.4 ± 3	24.9 ± 3	75.1 ± 3	20.4–38.6	19.6– 20.3	28.6

a Particulate organic material (POM) and particulate inorganic material (PIM) content of total suspended solids (TSS) for each month (*n* = 8) as determined by loss on ignition. The number of aggregates per milliliter, temperature, and salinity are ranges for the tidal cycle when samples were collected (i.e., flood and ebb tide values, respectively). N/A, not available.

**FIG 6:**
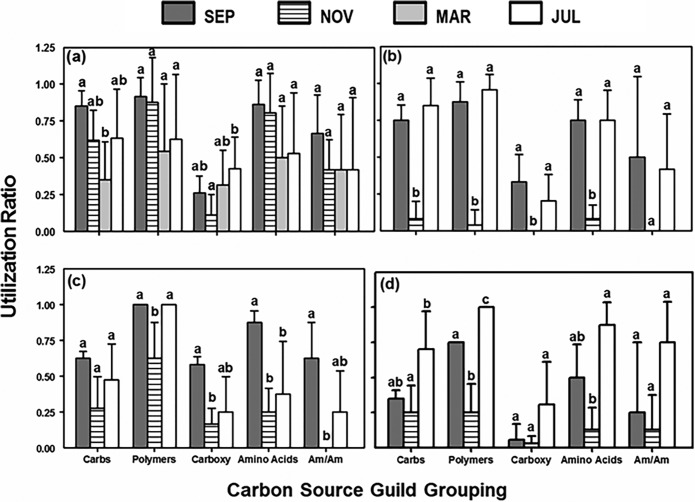
Carbon source utilization ratios of microbial communities, organized by guild groupings, for each of the sampled months. The carbon source guild groups were carbohydrates, polymers, carboxylic and acetic acids, amino acids, and amines/amides. Ratios were calculated by dividing utilization of each guild by total number of wells utilized (richness [*S*]). Sample types are mussel gut (a), oyster gut (b), aggregate (c), and aggregate-free seawater (d). Different letters denote significant differences in utilization ratios between months (two-way ANOVA, Tukey’s HSD, *P* < 0.05). Data are presented as means (plus SD) (*n* = 4 to 6 per carbon source per month).

**(iii) Guild groupings.** Significant temporal variation was found in the specific carbon sources (guilds) utilized by microbial communities of all sample types (one-way ANOVA on each guild; *P* < 0.05, df = 23 per bivalve, df = 11 per aggregate and AFSW; Fig. 6). Microbial communities of mussels demonstrated significant temporal differences in utilization of carbohydrates and carboxylic and acetic acids (Tukey’s HSD test; *P* < 0.05). All other guild groups were utilized in the same ratio regardless of the month (Fig. 6a). For oysters, microbial communities showed significant temporal differences in utilization of all guild groupings except for amines/amides (Tukey’s HSD test; *P* < 0.01; Fig. 6b). Microbial communities of aggregates demonstrated significant temporal differences in utilization of polymers, carboxylic and acetic acids, amino acids, and amines/amides (Tukey’s HSD test; *P* < 0.05; Fig. 6c). Utilization of carbohydrates was the same among months. For AFSW, utilization of carboxylic and acetic acids and amines/amides were not affected by month, but significant temporal effects were found for utilization of carbohydrates, polymers, and amino acids (Tukey’s HSD test; *P* < 0.05; Fig. 6d). In most cases where significant differences among months were found, utilization of the specific guild was lower in November compared to that of the other months.

### Total culturable heterotrophic bacteria and bivalve condition index.

Both month and sample type significantly affected the number of CFUs observed (two-way ANOVA, *P* < 0.05, df = 79). There was no significant effect of bivalve species on the number of CFUs except in November when there was a significant effect of month (Tukey’s HSD test, *P* < 0.05; Fig. S2a). Marine aggregates had significantly higher numbers of CFUs per milliliter than AFSW samples during all months (Fig. S2b). The length, width, and condition of each organism by month are presented in Table S2.

10.1128/mSphere.00730-19.2 FIG S2 (a and b) CFUs per milliliter of culturable heterotrophic bacteria in samples from mussel and oyster gut tissue (a) and aggregate and aggregate-free seawater (AFSW) (b). Capital letters designate significant differences between months, whereas lowercase letters designate significant differences between sample types within a month (two-way ANOVA, Tukey’s HSD, *P* < 0.05). When there are no letters, no significant effects were observed. The number of heterotrophic bacteria decreased in months with colder water temperatures for all sample types. Oysters and mussels had significant differences in the number of CFUs in their guts only in November. In all months, marine aggregate samples had significantly more CFUs per milliliter than AFSW. Data are presented as geometric means ± geometric SD (*n* = 4 to 6). Download FIG S2, PDF file, 0.1 MB. Copyright © 2019 Pierce and Ward. 2019 Pierce and Ward This content is distributed under the terms of the Creative Commons Attribution 4.0 International license.

10.1128/mSphere.00730-19.4 TABLE S2 Length, width, and condition of oysters and mussels from each sampling time point. Data are presented as means ± SD (*n* = 6 per bivalve per month). Download Table S2, PDF file, 0.08 MB. Copyright © 2019 Pierce and Ward. 2019 Pierce and Ward This content is distributed under the terms of the Creative Commons Attribution 4.0 International license.

### Video analysis, total suspended solids, and organic content.

The content of total suspended solids was largely inorganic, with more particulate organic material present during summer months (September and July; Table 2). Video analysis revealed a higher number of aggregates per milliliter on the ebb tide than the flood tide, and also in months with warmer water temperatures (Table 2).

## DISCUSSION

Results from this study revealed that two species of bivalves (Crassostrea virginica and Mytilus edulis), held together in the same environment, shared a core gut microbiome which is at least partially derived from the suspended-particle diet (e.g., marine aggregates). Although temporal variability was observed, animals harbored bacterial communities that were similar within each of the four sampling dates. These trends were maintained at the phylum and OTU level. Alpha diversity of the microbial communities, based on the Shannon diversity index, was not significantly different between summer and fall months for all sample types. Only samples in the winter (March) had significantly lower alpha diversity. A similar result was seen with the number of OTUs observed from each sample type.

The temporal trends of bivalve microbial communities have been explored minimally, but a few studies utilizing culture-independent methods have been conducted. Lokmer et al. observed alpha diversity in the hemolymph of Crassostrea gigas to be positively correlated with seawater temperature (58). Our previous research on C. virginica also showed temporal trends in microbial community structure correlated with seawater temperature (25). Although temporal trends were not studied, Vezzulli et al. found mussels and oysters held at a single site maintained gut microbiomes that were distinct, with some overlap (59). This supports findings from the current study and provides the only other direct comparison of mussel and oyster gut microbial communities.

Compared to studies on other marine organisms, bivalves from this study shared a high number of OTUs, 117. For example, tunicates from three distinct populations were found to share 35 OTUs and zebrafish from three different locations shared 21 OTUs (60, 61). In a study comparing wild and domesticated tiger shrimp, 18 OTUs were found to be shared (62). In this study, a total of 117 OTUs were shared among oysters and mussels from all seasons. Additionally, within a month, bivalves shared the largest proportion of total OTUs than to either of the other sample types. These findings are supported by our previous work (25), which showed high similarities among oyster microbiomes over space and time, with some seasonal variation. The sequencing results from the current study also align with characterization studies of other bivalve species (for a review, see reference 63).

Similar to the present study, Trabal et al. found that the most abundant bacterial phyla harbored by three species of adult oysters were *Bacteroidetes*, *Proteobacteria*, *Actinobacteria*, and *Firmicutes;* in particular, *Gammaproteobacteria* dominated many of the animals (23, 24). In fact, an abundance of *Gammaproteobacteria* has been commonly observed in *Crassostrea* spp. (64, 65). Fewer OTUs, however, were recovered by Trabal et al. compared to the current study which could be attributed to the use of different species of oysters and different hypervariable regions of the 16S rRNA gene (23, 24). King et al. also sequenced gut contents of the oyster, *C. virginica*, and found microbial communities dominated by *Mollicutes*, *Firmicutes*, *Proteobacteria*, *Chloroflexi*, and *Verrucomicrobia* (66). That study, however, lacked a temporal analysis and had a low sample size (*n* = 3). OTUs belonging to the *Mollicutes*, including the genus *Mycoplasma*, were found to be a member of the core bivalve gut microbiome in this study, and in particular have been consistently observed in abundance in bivalves (67–70). Although they evaluated different bivalve species, Vezzulli et al. also compared oysters and mussels and found sulfur metabolizers from the Deltaproteobacteria present in the gut (59). However, they found *Vibrio* spp. made up a large proportion of the gut microbiome, which we did not observe. Research with mussels of the genus *Brachidontes* also demonstrated the abundance of bacteria in the phyla *Proteobacteria*, *Firmicutes*, and *Tenericutes* (68), in line with results from the current study.

Many of those taxonomic groups present in the bivalve core were also shared with marine aggregates. While bivalves shared the largest proportion of OTUs with each other, about half of those were also shared with marine aggregates. Photosynthetic *Synechococcaceae* and the Planctomycete *Pirellulaceae* are likely food and are not colonizing and living in the guts of bivalves. Their presence is not surprising, given the ubiquity of these cyanobacteria in the marine environment and the association of planctomycetes with macroalgae (71), a component of marine aggregates. The high number of shared OTUs belonging to the *Desulfobulbaceae* and *Desulfobacteraceae* families are particularly of interest. These sulfur metabolizers are commonly observed in anaerobic environments, including mussels (59), and likely thrive both in the bivalve gut and in the anoxic core of aggregates (72). In fact, aggregates are a documented source of sulfide production (73) and may be a little-studied source of mercury methylation via sulfate-reducing bacteria (SRBs) (74). Pathogens were also shared among the aggregates and bivalves in this study, and the role of aggregates as a pathogen vector has been investigated by others (35, 39, 42, 56, 75). *Tenericutes* and *Spirochaetes* were not found in marine aggregates but were members of the bivalve gut microbial community. Bivalve association with these taxonomic groups has been well documented, especially spirochetes, which are considered oyster symbionts due to their association with the crystalline style in the gut (76). Together these results highlight and reinforce the idea that both extrinsic (particle diet) and intrinsic (host) factors are at play in shaping and mediating the bivalve microbiome.

On a functional level, oysters, aggregates, and AFSW showed significant seasonal variation, with carbon source utilization being largely undetected when water temperatures were lowest (March). In contrast, the richness, diversity, and evenness of mussel microbiomes did not change significantly with season. Utilization of most carbon guilds did not change over time, an indication of stable functional roles. The SIMPER analysis also showed that the diversity of the microbiome (at the phylum level) was stable temporally. Such stability could be a result of intrinsic factors of the host, rather than extrinsic factors of the food source (i.e., aggregates, AFSW) which did show temporal variation in taxonomic and functional diversity of the microbial communities. Mussels remain physiologically active during winter months (in contrast to oysters) with clearance rates (in liters per gram per hour) that can be similar between shifts in temperatures of 0.5 to 12°C (77). This physiological activity further supports the idea that mussels are not completely reliant on the environment to maintain their gut microbial communities, which can be decoupled from that of the suspended food source. These results highlight host species-specific differences, as microbial communities harbored in the oyster gut were less stable functionally and followed a trend similar to the suspended-particle diet.

Across all sample types, concentrations of culturable heterotrophic bacteria declined significantly during months when the seawater temperature was colder. This result is in agreement with other culture-dependent results of bivalve microbial communities (78–80). Although not as accurate as culture-independent methods, the concentrations of culturable heterotrophic bacteria from environmental samples did highlight the contrast between aggregates and aggregate-free seawater. Aggregate-associated bacterial communities are known to be highly diverse, and aggregates harbor communities which are “hot spots” of activity (36), which was corroborated by the marine agar results in this study.

Although the food source of bivalves may not be the only driving factor maintaining the gut microbiome, understanding the dynamic nature of bacterial communities of suspended particles will shed light on initial and continual colonization of particle-feeding invertebrates. To do so, characterizing aggregate-associated microbial communities is an important first step. A study of marine aggregates from the northern Adriatic Sea targeting the 16S rRNA gene found that the four most abundant groups in fall and winter were the *Bacteroidetes*, *Cyanobacteria*, and *Alpha*- and *Gammaproteobacteria* (47). Those phyla were three of the most abundant bacterial groups found in the current study, despite differences in the physical and chemical nature of the two water bodies. Additionally, there was little seasonal variation in the diversity of bacterial communities of aggregates (47), a finding similar to results of the current study. These results could be attributed to the stable environment aggregates provide. Characterization of unattached microbes in seawater has been more common and conducted on samples from different sites around the world. For example, Gilbert et al. found that *Alphaproteobacteria* was the most abundant class of bacteria in Atlantic waters off the coast of the United Kingdom in all seasons (81). A similar study of surface and bottom waters in the Adriatic Sea also found *Alphaproteobacteria* as the most dominant bacterial group (82). Taken together, these studies support the idea that marine aggregates harbor highly diverse microbial communities that are distinct from the surrounding seawater.

Overall, the current study saw functional differences among microbial communities, both seasonally and by sample type. The taxonomic structure of the microbial communities, however, remained largely stable, especially at the phylum level, between the two species of bivalves. Oysters and mussels harbored gut microbiomes that were more similar to one another than to the microbiomes of the environmental samples across all seasons, but especially within a month. The most abundant bacteria belonged to phyla commonly isolated from suspension-feeding bivalves by previous workers, despite some differences likely attributable to host species and geography. Core microbiomes have been found for a number of marine vertebrates and invertebrates, including zebrafish, corals, and tunicates (20, 60, 61), and highlight the prevalence of taxonomic redundancy in the microbial communities of these organisms. The high number of core OTUs shared by oysters and mussels compared to other marine organisms may be explained in part by their shared mode of suspension feeding. In many cases, however, the bacteria harbored by bivalves are distinct from the water column, and their conserved nature indicates that intrinsic factors shape and mediate the gut microbiome as well. Further work probing these factors in an experimental context, however, is needed to elucidate specific effects. Understanding the patterns and processes that mediate the diversity of microbial communities will lead to better insight into the intrinsic mechanisms that control bacterial-host interactions of aquatic invertebrates.

## MATERIALS AND METHODS

### Collection of samples.

Eastern oysters Crassostrea virginica (Gmelin, 1791) and blue mussels Mytilus edulis (Linnaeus, 1758) were held off the docks at the University of Connecticut Avery Point campus in Groton, CT, on Long Island Sound. Oysters were sourced from the Noank Aquaculture Co-op and held for a minimum of 2 months at Avery Point before sampling began. Mussels were collected from the docks at Avery Point. Oysters and mussels were selected that were of similar size, with oysters ranging from 8 to 10 cm in height and mussels ranging from 4 to 5 cm in length. The two species of bivalves were mixed together in baskets suspended from the dock at a depth of 1 m. Bivalves, marine aggregates, and seawater were collected for analyses four times—24 September (2013), 22 November (2013), 19 March (2014), and 1 July (2014)—using the following methods.

Marine aggregates were sampled using previously described methods (see reference 39 for figure). Briefly, seawater adjacent to the holding baskets was pumped into four individual 1-liter Imhoff cones that were held in a rack on the dock (39 for figure). Each cone was fitted with a sterile 15-ml Falcon tube at the bottom of its tapered end for collection of marine aggregates. Over a 60-min period, aggregates settled through the water column of each cone and into the Falcon tube. A 60-min settling time was chosen to separate large aggregates (>200 μm) from smaller particulates based on the settling velocity of different size aggregates (27) and the distance they settled in the cone (length, ca. 450 mm). After this period of time, 125 ml of seawater from the top of each cone was collected and placed into a cooler (operationally defined as aggregate-free seawater [AFSW]). AFSW samples were checked microscopically with a hemocytometer to confirm that no particulates >50 μm were present. Seawater in the settling cones was then removed, and a new 1-liter sample was added for settling. This process was repeated twice on the flood tide and twice on the ebb tide, with no samples collected within 2 h of slack tide. During periods of no sampling, Falcon tubes were stored at 4°C. Sample types were pooled over the course of the tidal cycle for each settling cone. At each collection time point, two 1-liter samples of seawater were taken to determine mass and organic content of total suspended solids (TSS). Temperature and salinity measurements were taken with a YSI meter during each collection time point.

Six individual oysters and mussels were sampled from the suspended baskets the morning following collection of water and marine aggregate samples. Sampled animals were immediately brought into the laboratory for processing and microbial community analysis (see below). Preliminary studies demonstrated that the day-to-day variation in the functional diversity of bacterial communities of aggregates was low, and this procedure ensured that aggregate and animal samples could be processed immediately after collection.

### Video analysis.

Each time seawater was collected, 5 min of underwater video footage was taken to enumerate suspended aggregates (Sony TRV99 camera in Ikelite housing) and analyzed as outlined by Lyons et al. (42). Still frames of aggregates were counted in ImageJ using the “analyze particles” function. Video footage allowed confirmation and quantification of the presence and size of marine aggregates in the bivalve’s immediate environment.

### Preparation of samples.

In the laboratory, aggregates in the Falcon tubes were allowed to settle for an additional 30 min, after which time the overlying water was removed. Marine aggregates were then diluted with 10 ml of phosphate-buffered saline (PBS) and disaggregated by shaking and vortexing (three times). AFSW from all sampling time points was pooled (total of 500 ml over the course of the tidal cycle per settling cone). AFSW was passed through a 20-μm polycarbonate filter, and the filtrate was then passed through a 0.2-μm polycarbonate filter. The 0.2-μm filter was placed in a sterile scintillation vial, and collected material (0.2 to 20 μm) was resuspended in 10 ml of PBS by vortexing (10 s). A total of four replicate samples of AFSW and aggregates were processed for each of the four sampling dates.

Bivalves were scrubbed to remove epibionts and rinsed with 70% ethanol. Oysters were dissected as outlined by Pierce et al. (25). Briefly, oysters were notched, the adductor muscle was severed, and biopsy punches were taken from the digestive gland and stomach (henceforth referred to as “gut”). For mussels, a small knife was inserted between the valves at the pedal gap, and the two adductor muscles were severed. The entire stomach and digestive gland were isolated and used (henceforth referred to as “gut”). Gut tissues were then homogenized and diluted in PBS as described by Pierce et al. (25). Due to size differences between oysters and mussels, only a portion of gut tissue was used from oysters, while the whole gut was sampled from mussels. This was done to ensure that equal amounts of tissue were used for comparison. Gut tissue from six oysters and six mussels were processed for each of the four sampling dates.

Subsamples of material collected from processed aggregates, AFSW, and homogenized oyster and mussel gut tissues from each sampling date were used immediately to inoculate EcoPlates and marine agar plates (see below). Additional subsamples (600 μl) of each of the collected material were stored at –20°C until processed for molecular analysis (see below).

### DNA extraction, PCR, and sequencing.

The Power Biofilm DNA Isolation kit (MoBio Laboratories Inc., Carlsbad, CA) was used to extract DNA from all samples (undiluted gut tissue, aggregates, AFSW) following the manufacturer’s instructions. The V4 hypervariable region of the 16S rRNA gene was amplified using previously designed 515F/806R primers with Illumina adapters and dual indices (see Table S3 in the supplemental material) (83). DNA was quantified using the Quant-iT PicoGreen kit (Invitrogen, ThermoFisher Scientific). Samples were amplified using Phusion High-Fidelity PCR master mix (New England BioLabs). The PCR mixture was incubated at 94°C for 3 min. The mixture was then subjected to 30 cycles of PCR, with 1 cycle consisting of 45 s at 94.0°C, 60 s at 50.0°C, and 90 s at 72.0°C, followed by a final extension step at 72.0°C for 10 min.

10.1128/mSphere.00730-19.5 TABLE S3 Primers used in this study, including Illumina adapter, barcode, pad, and linker. Primer sequences and lengths are from Caporaso et al. (83). Download Table S3, PDF file, 0.08 MB. Copyright © 2019 Pierce and Ward. 2019 Pierce and Ward This content is distributed under the terms of the Creative Commons Attribution 4.0 International license.

Libraries were prepared and sequenced on an Illumina MiSeq at The Microbial Analysis and Research Services (MARS) facility (University of Connecticut). Libraries were prepared as described by Caporaso et al. using a 2 × 250-bp paired-end protocol (84).

### Community-level physiological profiling (Biolog EcoPlates).

The EcoPlate contains a triplicate series of 31 unique carbon sources (10 carbohydrates, 9 carboxylic acids, 4 polymers, 6 amino acids, and 2 amines/amides) plus a control well (with no substrate). Each well additionally contains a tetrazolium violet dye which is activated in the presence of microbial respiration. The utilization of a carbon source is evaluated by comparing the optical density of each well to a threshold value (0.25).

Biolog EcoPlates assess microbial community functional diversity, defined as the potential catabolic activity, by evaluating the number (substrate richness *S*) and pattern of carbon source utilization by the community (85). Although the plates assess only one microbial function (carbon metabolism), functional diversity is the term commonly used in the literature to describe results of this method (25, 35, 86–88).

Aliquots (150 μl) of material collected from AFSW, aggregates, and oyster and mussel tissues were inoculated into EcoPlates, incubated in the dark at 21°C, and read as outlined by Pierce et al. (25). As described above, samples were diluted with PBS to minimize the false-positive effects of divalent cations while maximizing bacterial community diversity (25, 88).

Absorbance values from 168-h readings were chosen as the endpoint and corrected to control for any inherent color change from carbon sources in the inoculum as outlined by Insam and Goberna (89). Calculations of richness (*S*), the number of substrates utilized by the microbial community of the sample, were determined as outlined by Zak et al. (85) and Lyons et al. (35). The Shannon diversity index (*H*′ = −Σ*p_i_*ln *p_i_* where *p_i_* is the relative abundance of the absorbance value of each well – individual well absorbance/sum of all absorbance values), and evenness (*E* = *H*′/ln *S* where *H*′ is the Shannon diversity index and *S* is the richness) were calculated using wells in which corrected absorbance values were >0.25. Guild groupings by substrate type (carbohydrate, carboxylic acid, polymer, amino acid, and amines/amides) were assigned following the recommendations of Zak et al. (85) and Weber and Legge (90). Utilization ratios for each grouping were calculated based on the number of wells utilized divided by the total number of wells possible (31) per plate.

### Total culturable heterotrophic bacteria.

Difco Marine Agar 2216 (Becton, Dickinson and Co.) was used to enumerate marine heterotrophic bacteria. Aliquots (100 μl) of material collected from diluted bivalve gut tissue, marine aggregates, and AFSW samples were spread onto agar plates and incubated at the ambient seawater temperature at the time of collection. Plates were read and recorded every 24 h for 5 days using a colony counter (Quebec Reichert, Inc.). The total number of CFUs was used from the final day of reading.

### Bivalve condition index.

Dry tissue and shell weights were determined and used to calculate individual condition indices (CI), an indication of the overall health of the animal. CI was calculated using the following equation from Crosby and Gale (91):

$$\text{CI}=\frac{\text{soft tissue weight}\times \text{1,000}}{\text{total weight}-\text{shell weight}}$$

where soft tissue weight was dry weight and all weights were in grams.

The height (oysters), length (mussels), and width of the shell of each animal were measured using calipers and recorded.

Because of the smaller size of the mussel gut compared to the oyster, no gut tissue remained for weighing after microbial analyses. To account for the weight lost by removal of gut tissue, the guts of a second set of mussels, representative of the population sampled (i.e., similar length and width), were isolated, dried, and weighed. The mean dry weight of these gut tissues was added to the dry weight recorded for mussels from the original experiment to ensure accurate condition index calculations.

### Total suspended solids and organic content.

As described above, two 1-liter samples of seawater were taken to determine the mass and organic content of total suspended solids (TSS). The eight replicate seawater samples (1 liter) from each sampling date were vacuum filtered onto precombusted, preweighed GF/C filters (Whatman; 47 mm). Filters were rinsed with isotonic ammonium formate to remove salts and dried to a constant weight at 70°C, and TSS were recorded. The filters were then combusted at 450°C for 2 h and reweighed to determine the organic content of the samples (92, 93).

### Amplicon sequencing analysis.

Read pairs were merged, quality and length filtered prior to being demultiplexed and analyzed by using the Quantitative Insights Into Microbial Ecology (QIIME, v. 1.8.0) pipeline (94). Greengenes (13_8 release) (95) was used as a reference database for the taxonomic assignment of operational taxonomic units (OTUs) based on 97% sequence similarity. The data set was filtered to remove singletons, doubletons, and sequences classified as mitochondria and chloroplasts. Core microbiomes were defined as the OTUs present in 95% of samples. Alpha and beta diversity were determined in QIIME using the alpha_diversity.py and beta_diversity.py scripts, respectively. Alpha diversity metrics “shannon” and “osd” were run on subsampled data at a sequencing depth of 15,602. The beta diversity metric “bray_curtis” was run on nonsubsampled data.

### Statistical analysis.

QIIME output files were additionally analyzed using the phyloseq package (v. 1.19.1) in R and Statistical Analysis of Metagenomic Profiles (STAMP, v. 2.1.3). A one-way analysis of variance (ANOVA) was performed to examine the effect of sample type on bacterial abundance at multiple taxonomic levels (STAMP v.2.1.3). If a significant effect was detected, a Tukey-Kramer post hoc test was applied. Significance was assessed using an alpha level of 0.05.

Permutational multivariate analysis of variance (PERMANOVA) was conducted in R (adonis function, vegan package v. 2.4-4) using Bray-Curtis OTU-based distance matrices to test the effect of the factors time (month) and sample type on the microbial community structure. Homogeneity of variance was evaluated with the betadisper function which utilizes a multivariate analogue of Levene’s test. Only results that did not violate the homogeneity assumption of the PERMANOVA test were interpreted as the most reliable, even given an unbalanced design (96).

Two-way analysis of variance tests were performed to examine the effects of the two independent variables, month and sample type, on Shannon diversity index and number of OTUs observed calculated from 16S rDNA sequences. Due to the small sample size in some months and sample types, two-way crosses could not be conducted. This is because amplification of environmental samples from winter months was largely unsuccessful. There was additionally difficulty with inhibitors from bivalve gut tissue, which contributed to an unbalanced sample design. Individual one-way ANOVAs were run instead. A one-way ANOVA was performed to examine the effect of sample type on bacterial abundance at multiple taxonomic levels (STAMP, v. 2.1.3).

Due to amplification issues, sample sizes for sequencing data were as follows: for mussels, *n* = 2 (September), *n* = 4 (November), *n* = 1 (March), and *n* = 6 (July); for oysters, *n* = 4 (September), *n* = 1 (November), *n* = 0 (March), and *n* = 4 (Jul); for aggregates, *n* = 3 (September), *n* = 4 (November), *n* = 3 (March), and *n* = 1 (July); for AFSW, *n* = 0 (September, November, and March) and *n* = 3 (July). Statistical analyses using EcoPlate and marine agar data had sample sizes that included all samples (bivalves [*n* = 6 per month], environmental [*n* = 4 per month]).

Two-way ANOVAs were performed to examine the effects of the two independent variables, month and sample type, on carbon source utilization (richness), Shannon diversity index, and evenness calculated from EcoPlate data, as well as CFUs of heterotrophic bacteria. A one-way ANOVA was used to examine the effect of month on carbon utilization ratios by guild grouping for each sample type.

For all ANOVAs, the general linear model (GLM) (Systat13) procedure was used, and if significant effects were detected, a Tukey’s honestly significant different (HSD) post hoc test was performed to examine the differences between the levels of the independent variables. All data were tested for normality and homoscedasticity prior to analyses. Data that did not meet these assumptions were transformed (arcsine or log) to improve normality and homogeneity of variance. Significance was assessed using an alpha level of 0.05.

SIMPER analysis was run using the program PRIMER (v.6). For EcoPlate analysis, corrected absorbance values were used, whereas 16S amplicon analysis used abundance data at the phylum level. One-way and two-way crossed SIMPERs were run with a cutoff of 90%, using the Bray-Curtis similarity resemblance measure.

### Data availability.

Raw sequencing reads are available for download from the NCBI Sequence Read Archive under BioProject accession number PRJNA386685.
