# Supplementary material for: Gut Microbiomes of the Eastern Oyster (Crassostrea virginica) and the Blue Mussel (Mytilus edulis): Temporal Variation and the Influence of Marine Aggregate-Associated Microbial Communities
Source: mSphere. 2019 Dec 11;4(6):e00730-19. doi: 10.1128/mSphere.00730-19 (PMC6908423; doi:10.1128/mSphere.00730-19)
Supplement: TABLE S1 [file mSphere.00730-19-st001.pdf]

16S - Phylum Level	Avg. Within Group Similarity							Avg. Dissimilarity Between Sample Types			
			**Sep**	**Nov**	**Mar**	**Jul**		**Mussel**	**Oyster**	**Marine Aggregate**	**AFSW**
**Mussel**	88.2		89.8	90.5	n=1	87.2		-	18.3	27.1	
**Oyster**	87.0		84.1	87.4	-	87.2			-	31.9	
**Marine Aggregate**	83.9		89.8	83.5	78.8	n=1				-	
**AFSW**	92.3		-	-	-	92.3					-

EcoPlates	Avg. Within Group Similarity							Avg. Dissimilarity Between Sample Types			
			**Sep**	**Nov**	**Mar**	**Jul**		**Mussel**	**Oyster**	**Marine Aggregate**	**AFSW**
**Mussel**	52.0		87.3	63.2	47.1	67.0		-	64.4	62.5	71.2
**Oyster**	43.7		80.5	22.0	35.1	76.2			-	61.8	67.1
**Marine Aggregate**	54.6		84.5	46.8	34.3	74.1				-	57.3
**AFSW**	37.9		58.8	15.7	25.9	79.1					-

16S - Phylum Level	Avg. Dissimilarity Between Months by Sample Type					
	**Sep v. Nov**	**Sep v. Mar**	**Nov v. Mar**	**Sep v. Jul**	**Nov v. Jul**	**Mar v. Jul**
**Mussel**	15.2	24.7	21.8	18.6	19.2	24.8
**Oyster**	13.2	-	-	17.1	18.0	-
**Aggregate**	13.6	26.3	33.2	20.0	23.0	27.2
**AFSW**	-	-	-	-	-	-

EcoPlates	Avg. Dissimilarity Between Months by Sample Type					
	**Sep v. Nov**	**Sep v. Mar**	**Nov v. Mar**	**Sep v. Jul**	**Nov v. Jul**	**Mar v. Jul**
**Mussel**	30.2	43.0	50.9	32.6	41.5	43.8
**Oyster**	79.6	77.2	71.6	22.6	78.7	77.1
**Aggregate**	52.1	72.6	69.1	26.0	52.7	68.0
**AFSW**	71.5	76.7	83.9	40.4	73.4	77.0
