# Supplementary figures and images for: Gut Microbiomes of the Eastern Oyster (Crassostrea virginica) and the Blue Mussel (Mytilus edulis): Temporal Variation and the Influence of Marine Aggregate-Associated Microbial Communities
Source: mSphere. 2019 Dec 11;4(6):e00730-19. doi: 10.1128/mSphere.00730-19 (PMC6908423; doi:10.1128/mSphere.00730-19)

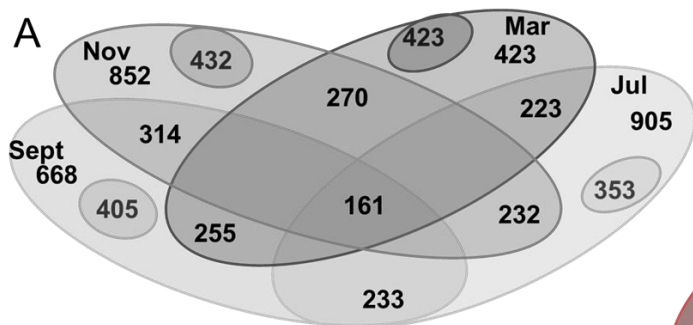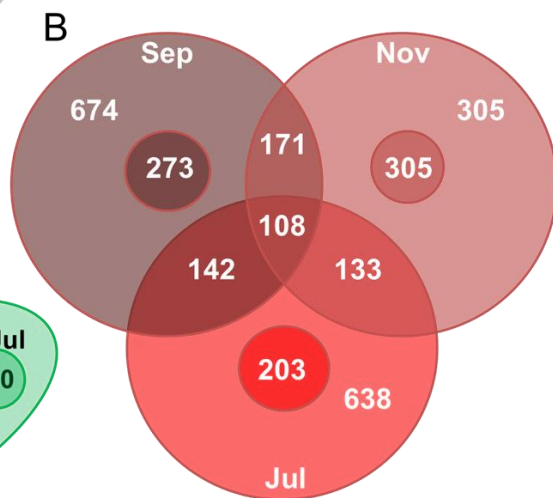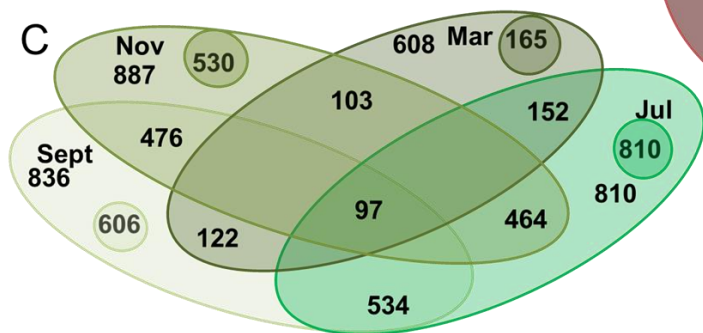

Supplement: FIG S1 [file mSphere.00730-19-sf001.pdf]

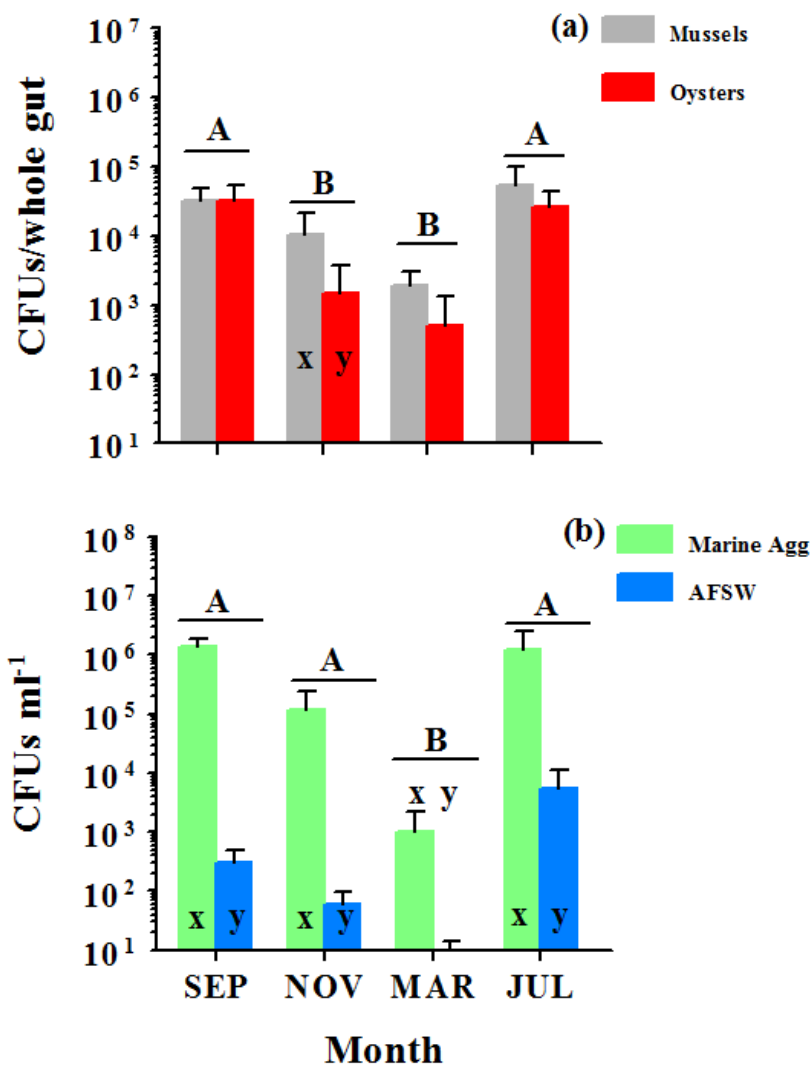

Supplement: FIG S2 [file mSphere.00730-19-sf002.pdf]
