# Supplementary material for: Gut Microbiomes of the Eastern Oyster (Crassostrea virginica) and the Blue Mussel (Mytilus edulis): Temporal Variation and the Influence of Marine Aggregate-Associated Microbial Communities
Source: mSphere. 2019 Dec 11;4(6):e00730-19. doi: 10.1128/mSphere.00730-19 (PMC6908423; doi:10.1128/mSphere.00730-19)
Supplement: TABLE S2 [file mSphere.00730-19-st002.pdf]

**Month**	**CI**		**length (mm)**		**width (mm)**	
	Oyster	*Mussel*	*Oyster*	*Mussel*	*Oyster*	*Mussel*
**SEP**	60.5 ± 10	68.2 ± 6.1	96 ± 7.2	42.7 ± 1.7	80.5 ± 10.9	24 ± 1.4
**NOV**	51.4 ± 8.6	59.8 ± 18.6	95.7 ± 6.6	41.6 ± 2	76.4 ± 8.2	22.2 ± 0.4
**MAR**	54 ± 11.7	105.8 ± 23.4	91.6 ± 4.4	46 ± 2.1	76.1 ± 8.4	24 ± 1.4
**JUL**	48.1 ± 15.4	92 ± 20.5	103 ± 8.6	49.7 ± 1.6	83.1 ± 11.9	25.3 ± 1.3
