# Supplementary material for: Gut Microbiomes of the Eastern Oyster (Crassostrea virginica) and the Blue Mussel (Mytilus edulis): Temporal Variation and the Influence of Marine Aggregate-Associated Microbial Communities
Source: mSphere. 2019 Dec 11;4(6):e00730-19. doi: 10.1128/mSphere.00730-19 (PMC6908423; doi:10.1128/mSphere.00730-19)
Supplement: TABLE S3 [file mSphere.00730-19-st003.pdf]

Name	Primer Sequence 5’ -> 3’	Length
515F	AATGATACGGCGACCACCGAGATCTACAC TATGGTAATT GT GTGCCAGCMGCCGCGGTAA	60
806R	CAAGCAGAAGACGGCATACGAGAT XXXXXXXXXXXX AGTCAGTCAG CC GGACTACHVGGGTWTCTAAT	68
